# Structural and Mechanistic Bases of Nuclear Calcium Signaling in Human Pluripotent Stem Cell-Derived Ventricular Cardiomyocytes

**DOI:** 10.1155/2019/8765752

**Published:** 2019-04-01

**Authors:** Sen Li, Wendy Keung, Heping Cheng, Ronald A. Li

**Affiliations:** ^1^Stem Cell & Regenerative Medicine Consortium, LKS Faculty of Medicine, University of Hong Kong, Hong Kong; ^2^Dr. Li Dak-Sum Research Centre, The University of Hong Kong, Pokfulam, Hong Kong; ^3^Institute of Molecular Medicine, Peking University, Beijing, China; ^4^Ming-Wai Lau Centre for Reparative Medicine, Karolinska Institutet, Hong Kong

## Abstract

The loss of nonregenerative, terminally differentiated cardiomyocytes (CMs) due to aging or diseases is generally considered irreversible. Human pluripotent stem cells (hPSCs) can self-renew while maintaining their pluripotency to differentiate into all cell types, including ventricular (V) cardiomyocytes (CMs), to provide a potential unlimited *ex vivo* source of CMs for heart disease modeling, drug/cardiotoxicity screening, and cell-based therapies. In the human heart, cytosolic Ca^2+^ signals are well characterized but the contribution of nuclear Ca^2+^ is essentially unexplored. The present study investigated nuclear Ca^2+^ signaling in hPSC-VCMs. Calcium transient or sparks in hPSC-VCMs were measured by line scanning using a spinning disc confocal microscope. We observed that nuclear Ca^2+^, which stems from unitary sparks due to the diffusion of cytosolic Ca^2+^ that are mediated by RyRs on the nuclear reticulum, is functional. Parvalbumin- (PV-) mediated Ca^2+^ buffering successfully manipulated Ca^2+^ transient and stimuli-induced apoptosis in hPSC-VCMs. We also investigated the effect of Ca^2+^ on gene transcription in hPSC-VCMs, and the involvement of nuclear factor of activated T-cell (NFAT) pathway was identified. The overexpression of Ca^2+^-sensitive, nuclear localized Ca^2+^/calmodulin-dependent protein kinase II *δ*_B_ (CaMKII*δ*_B_) induced cardiac hypertrophy through nuclear Ca^2+^/CaMKII*δ*B/HDAC4/MEF2 pathway. These findings provide insights into nuclear Ca^2+^ signal in hPSC-VCMs, which may lead to novel strategies for maturation as well as improved systems for disease modeling, drug discovery, and cell-based therapies.

## 1. Introduction

Human pluripotent stem cells (hPSCs) can self-renew while harboring pluripotency to differentiate into various cell types including ventricular (V) cardiomyocytes. Directed cardiac differentiation protocols have been established to generate hPSC-VCMs in large quantities [1] which provide an unlimited ex vivo source of hPSC-CMs for disease modeling and drug discovery as well as cell-based heart therapies. Given its physiological importance in excitation-contraction coupling, cytosolic Ca^2+^ signals in hPSC-CMs have been extensively studied [2–4]. By contrast, although nuclear Ca^2+^ signaling is known to affect a range of cellular physiological processes [5] from cell proliferation [6], meiosis reinitiation [7], gene transcription [8, 9], cell protection [10] to cardiac hypertrophy and heart failure [11], this intriguing process has only recently been characterized in rat cardiomyocytes [5]and is poorly defined in hPSC-VCMs. Here, we sought to address the gap by comprehensively investigating both the global and local nuclear Ca^2+^ signals in hPSC-VCMs. We tested the hypothesis that nuclear Ca^2+^ signaling plays a pivotal role in human cardiac differentiation and maturation of hPSC-VCMs by studying in detail its structural and mechanistic bases in relation to those of the cytosolic Ca^2+^ pathway by drawing comparison between these cells and adult cardiomyocytes.

## 2. Methods

### 2.1. Cardiac Differentiation and Selection of Ventricular Cardiomyocytes

Trypsin-tolerated human embryonic stem cells (hESCs) line HES2 was cultured and maintained in mTeSR™1 on Matrigel (BD, Franklin Lakes, NJ)-coated plates. Directed cardiac differentiation was carried out according to our previously published method [12]. In brief, HES2 hESCs (Wicell, Madison, WI) were dissociated into single cells using Accutase (Invitrogen, Carlsbad, CA) at day 0 of differentiation and resuspended in ultra-low attachment plates in mTeSR™1 (Stem Cell Technologies, Vancouver, Canada) supplemented with Matrigel™ (40 *μ*g/mL, BD Biosciences, San Jose, CA), BMP4 (1 *η*g/mL, Invitrogen), and Rho kinase (ROCK) inhibitor (10 *μ*M, R&D, Minneapolis, MN) under hypoxic condition (5% O_2_). At day 1, mTeSR™1 was replaced with StemPro-34 (Invitrogen) containing BMP4 (10 *η*g/mL), human recombinant activin A (10 *η*g/mL, Invitrogen), ascorbic acid (50 *μ*g/mL, Sigma, St. Louis, MO), GlutaMAX (2 mM, Gibco, Carlsbad, CA), and 1% Penicillin/Streptomycin (Gibco). At day 4, the cytokines were replaced with IWR-1 (5 *μ*M, Enzo, Farmingdale, NY). At day 8, cells were transferred to normoxic condition and maintained with StemPro-34 supplemented with ascorbic acid. Twenty to thirty days after directed cardiac differentiation, beating clusters were dissociated into single cells by trypsin. The day following cell dissociation, transduction was performed using lentiviral particles generated by cotransfecting 293FT cells with the packaging plasmid (pCMV-Delta 8.91), envelope VSVG plasmid (pMD.G), and ventricle selection plasmid (pMLC2v-tdTomato-T2A-Zeo or pMLC2v-GFP-T2A-Zeo) and concentrated from 293FT cell culture medium by PEG-*it* (SBI, Mountain View, CA). Zeocin (Invitrogen) treatment (300 *μ*g/mL, one week) was used to select hESC-VCMs three days after transduction.

### 2.2. Ca^2+^ Measurements

Zeocin-selected hESC-VCMs, seeded on MatTek dishes with glass bottom (MatTek, Ashland, MA), were washed once with Tyrode solution containing 140 mM NaCl, 5 mM KCl, 1 mM MgCl_2_, 1 mM CaCl_2_, and 10 mM D-glucose buffered with 10 mM HEPES at pH 7.4. Then cells were loaded with Fluo-4 AM at a concentration of 4 *μ*M for 30 min at 37°C. Zeiss LSM 700 inverted confocal microscope equipped with solid state lasers (405 nm for Hoechst dye, 488 nm for Fluo-4 AM, and 555 nm for tdTomato) and 63x oil objective with 1.4 numerical aperture was used to obtain line scanning and 2D imaging results. For recording nuclear Ca^2+^ signal, scanning line was selected to cross cytosol and the center of nucleus (c.f.  Figure S1A). To exclude any potential differences resulting from different Ca^2+^ dye characteristics in the nucleus and the cytosol [13], caged Ca^2+^ (NP-EGTA) [14] was loaded into tdTomato-positive hESC-VCMs, with the nuclear area labeled by mitochondrial dye as described [15] (Figure S2). When the line scanning figure was obtained by simultaneously uncaging Cage-Ca^2+^ and recording Fluo-4 fluorescence, the result indicated that nuclear and cytosolic Ca^2+^ signal had similar kinetics (Figure S2). For data analysis, rise time was defined as time required from 25% to 75% peak amplitude in the rise phase of Ca^2+^ signal while decay time was the time required from 75% to 25% peak amplitude in the decay phase of Ca^2+^ signal. For Ca^2+^ spark measurement, hESC-VCMs were paced at 0.5 Hz for 10 times, and then the electrical stimulation was stopped. Line scanning was repeated 1000 times with a time interval of 3.055 ms. For measurement involving 2-photon laser, Zeiss LSM 710 upright confocal microscope equipped with MaiTai HP tunable 2-photon (690-1040 nm) was employed. Intact nuclei were isolated according to published methods [16]. Briefly, hESC-VCMs were detached by trypsin and homogenized by a Dounce glass homogenizer on ice with ice-cold buffer A (0.25 M sucrose, 5 mM MgCl_2_, and 10 mM tris-HCL, pH 7.4). Then the disrupted cells were centrifuged at 600 g for 10 min at 4°C. The pellet was washed once with buffer A and resuspended in ice-cold buffer B (2.0 M sucrose, 1 mM MgCl_2_, and 10 mM Tris-HCL, pH 7.4). Centrifugation at 16000 g at 4°C for 30 min was performed, and the resulting pellet of isolated nuclei was resuspended in buffer A and stored at -70°C. Ca^2+^ in nucleoplasm or nuclear envelope was measured as described [17]. In brief, isolated nuclei were loaded with 20 *μ*M calcium green dextran (potassium salt; MW 10000) for 1 h at 4°C in internal solution containing 100 mM K-aspartate, 20 mM KCl, 1 mM MgCl_2_, 0.4 mM EGTA, 0.18 mM CaCl_2_, 4% Dextran, 1 mM ATP, 20 mM creatine phosphate, 20 U/mL creatine kinase, and 20 mM Hepes with pH 7.2 adjusted by HCl/KOH. After washing, Ca^2+^ measurement was performed in internal solution using Zeiss LSM 700 confocal microscope. For Ca^2+^ measurement in nuclear envelope, 20 *μ*M membrane-permeant Fluo-5N AM was used for loading at room temperature for 3 hours. After dye loading, Zeiss LSM 700 confocal microscope equipped with 488 nm solid state lasers was employed for the measurement. Pharmacological agents were applied as indicated. Ryanodine and FPL 64176 were purchased from Tocris (Bristol, UK). Fluo-4 AM and ER tracker were purchased from Invitrogen. Other drugs were obtained from Sigma. All recordings were performed at 37°C. For electrical pacing, field stimulation was provided by a voltage generator (Digitimer, UK) at different frequencies as indicated.

### 2.3. Immunostaining

Cells were washed twice with phosphate-buffered saline (PBS) and fixed with 4% paraformaldehyde in PBS for 15 min at room temperature. After washing three times with PBS, hESC-VCMs were blocked and permeabilized in PBS containing 1% bovine serum albumin (BSA), 5% goat serum, and 0.1% Triton X-100 for 30 min at room temperature. Then cells are incubated with primary antibodies against Lamin B (Santa Cruz Biotechnology, Santa Cruz, CA), RyR2 (Sigma), IP_3_Rs (Millipore, Billerica, MA), NFATc3 (Santa Cruz Biotechnology), ANP (Santa Cruz), CaMKII (Santa Cruz), His tag (NeuroMab, Davis, CA), or HDAC4 (Santa Cruz Biotechnology) at 4°C overnight. Alexa Fluor-488 IgG (Invitrogen) or Alexa Fluor-647 IgG (Invitrogen) was used as secondary antibodies. Nuclei were stained with DAPI (Invitrogen). Zeiss LSM 700 inverted confocal microscope was used to capture images.

### 2.4. Western Blotting

Proteins were extracted from antibiotic-selected cells with ice-cold denaturing cell extract buffer (Invitrogen), and the lysates were passed through a 21-gauge needle several times to disperse large aggregates. Protein assay was performed with Pierce BCA protein assay kit (Thermo Scientific, Rockford, IL), and proteins (40 *μ*g per lane) were then diluted in NuPAGE LDS Sample Buffer (Invitrogen) and subjected to electrophoresis on NuPAGE Novex 12% Bis-Tris Gels (Invitrogen). Next, proteins were transferred to PVDF membrane (Bio-Rad, Hercules, CA). Blocking was performed in PBS with 5% milk and 0.1% Tween 20. Incubation with primary antibodies against PV (Sigma), Hsp 90 (BioVision, Milpitas, CA), Oct-1 (Enzo), IP_3_Rs (Millipore), RyR2 (Sigma), or phospho-CaMKII (Cell Signaling, Danvers, MA) was carried on in 4°C overnight. After washing with PBS containing 0.1% Tween 20, the blots were probed with secondary antibodies for detection by chemiluminescence. Cytosolic and nuclear protein extracts were prepared with NE-PER nuclear and cytoplasmic extraction reagents (Thermo Scientific) according to manufacturer's protocol.

### 2.5. Subcloning

Adenovirus-based PV constructs were obtained from Addgene [6], and AccuPrime™ Pfx SuperMix (Invitrogen) was used to perform PCR reactions to amplify DsRed (control), PV-NES-DsRed, PV-NLS-DsRed, and PV-NLS-CD-DsRed fusion gene from these constructs. Subcloning sites NheI and BamHI were introduced by PCR primers. The PCR products were subcloned into pLV-CMV-MCS- (multiple cloning site-) EF1*α*-Puro (SBI) as backbone for expressing PV fusion proteins in hESC-VCMs. For stem cells, another backbone pLV-EF1*α*-MCS-T2A-Puro (SBI) was used. Lentivirus packaging and cell transduction were performed as described in the previous section. Three days after transduction, puromycin (Invitrogen) selection (3 *μ*g/mL, three days) was performed. The wild-type and constitutively active CaMKII*δ*_c_ plasmids were generous gifts from Prof. Rui-Ping Xiao's lab [18], and the genes of interest were subcloned into pLV-EF1*α*-MCS-IRES-Neo (SBI) backbone. CaMKII*δ*_B_ was generated by inserting the 33 bp (11 amino acids contain nuclear localization sequence) which represents the only different sequence between CaMKII*δ*_B_ and *δ*_c_ [19]. Lentivirus packaging and cell transduction were performed the same way as described in the previous section. Three days after transduction, G418 (Invitrogen) selection (500 *μ*g/mL, one week) was performed. The HDAC4 construct was from Addgene [20], and subcloning was performed to transfer HDAC4 into the pLV-EF1*α*-MCS-IRES-Neo (SBI) backbone. Lentivirus packaging, cell transduction, and selection were performed as described in the previous section.

### 2.6. Luciferase Activity Measurement

Cells were cotransfected with NFAT-luc plasmid (Promega, Madison, WI) and Renilla luciferase control plasmid (Promega) with a ratio of 50 : 1. The indicated drugs were applied 24 hours after transfection for 18 hours. Then, the cells were harvested with lysis buffer, and NFAT activity was measured by a dual-luciferase reporter assay system (Promega), following the protocol from the manufacturer. Signal was captured by TECAN Infinite® 200 plate reader (Tecan, Switzerland).

### 2.7. Spontaneous Differentiation and Q-RT-PCR

At day 0 of differentiation, HES2 hESCs were detached by dispase and resuspended in low attachment plates in EB medium containing 2 mM L-glutamine (Gibco), 1% Penicillin/Streptomycin (Gibco), 20% knockout serum replacer (Gibco), MEM nonessential amino acids (Gibco), and 55 *μ*M 2-Mercaptoethanol (Gibco) in DMEM (Gibco). EB medium was used during the whole differentiation process. RNA from a 14-day embryoid body was extracted by RNeasy Plus Mini Kit (Qiagen, Netherlands), and cDNA was synthesized by QuantiTect Reverse Transcription Kit (Qiagen) according to product protocol. Q-RT-PCR was performed with KAPA SYBR fast qPCR kit (Kapa Biosystems, Woburn, MA) according to manufacturer's manual. GAPDH was used as the internal control. The primers used for the three germ layer makers are Sox17 (forward 5′CGCTTTCATGGTGTGGGCTAAGGACG, reverse 5′TAGTTGGGGTGGTCCTGCATGTGCT), Hand1 (forward 5′CCACCCTTTTGGAGCGAATT, reverse 5′AATTAGAGAAGACGGCGTCGG), Igf2 (forward 5′CATCTCCCTTCTCACGGGAAT, reverse 5′GTTGCTATTTTCGGATGGCC), Pax6 (forward 5′CCAGCTTCACCATGGCAAAT, reverse 5′GGCAGCATGCAGGAGTATGAG), and cTnT (forward 5′AAGAGGCAGACTGAGCGGGAAA, reverse 5′AGATGCTCTGCCACAGCTCCTT). For the examination of CaMKII*δ*_B_ function, the primers for involved genes are Nur77 (forward 5′AGCATTATGGTGTCCGCACAT, reverse 5′TTGGCGTTTTTCTGCACTGT), ANP (forward 5′CAGACCAGAGCTAATCC, reverse 5′CTCACTGAGCACTTGT), BNP (forward 5′CCCACAGGTGTCTGGAAGTC, reverse 5′GGTCCATCTTCCTCCCAAAG), and MYH6 (forward 5′CAGCACAGAGCTCTTCAAGC, reverse 5′GTCCGAGATTTCCTCCTGAA). Results represent three independent repeats.

### 2.8. Microtissue Generation and Force Measurement

Polydimethylsiloxane (PDMS, Sylgard 184, Dow Corning)-microfabricated tissue gauge substrates were molded from the SU-8 masters, with embedding fluorescent microbeads (Fluoresbrite 17147, Polysciences Inc.) into the cantilevers to accommodate computerized cantilever deflection tracking for force measurement as previously described [21]. Cardiac clusters were digested into single cell by trypsin, resuspended in M199 medium (Gibco) containing 1.5 mg/mL collagen I (BD Biosciences) and 0.5 mg/mL fibrinogen (Sigma), added onto the substrates (1.2 million cells per mold), and degassed by vacuum on ice. Centrifugation was applied to settle the cells into micropatterned wells with around 500 cells per well. hvCMTs were transduced with lentiviruses at day 2, and measurements were made at day 5. Brightfield and fluorescent images were taken every 25 ms by Perkin Elmer spinning confocal microscope at 37°C. The displacement of the cantilevers was tracked by an ImageJ plugin SpotTracker [22].

### 2.9. Data Analysis

ImageJ was used to convert line scan figures into curves. Cytosolic and nuclear Ca^2+^ rise was normalized by scaling between 0 and 1 as indicated. Rise time is quantified as time needed from 25% peak amplitude to 75% peak amplitude while decay time is quantified as time needed from 75% peak amplitude to 25% peak amplitude. Results were expressed as mean ± SE (standard error). Student's *t*-test was used to determine the statistical significance, and *p* values < 0.05 (^∗^) or 0.01 (^∗∗^) were deemed statistically significant.

## 3. Results

### 3.1. Nuclear Ca^2+^ Signal in hESC-VCMs during Ca^2+^ Wave and Transient

For measuring nuclear Ca^2+^ transients or sparks, line scanning was performed across Hoechst 33342-labeled nuclei [23] (Figure S1A-C). After recording the Fluo-4 fluorescence, the excitation wavelength was changed for recording the preloaded Hoechst 33342 fluorescence. As such, the exact boundary of the nuclear area could be identified for measuring any changes in nuclear Ca^2+^ [24].  Figure S1D shows that propagating Ca^2+^ waves crossing the nucleus (white circle) could be observed in hESC-VCMs when _ext_[Ca^2+^] was increased to 10 mM. These were completely abolished by ryanodine (Figure S1E). In the line scan mode, nuclear Ca^2+^ signals displayed a lower propagating velocity (Figure S1F). When quantified, slowed rise and decay were also noticeable (Figure S1G).

**Figure 1:**
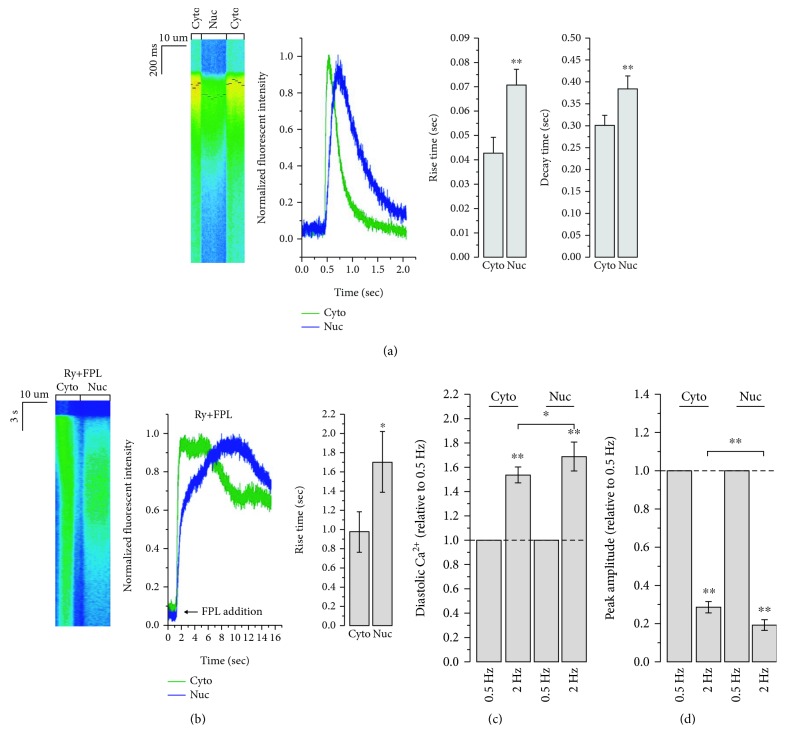
Nuclear Ca^2+^ signaling during Ca^2+^ transients. (a) Nuclear Ca^2+^ signal showed delayed kinetics compared to cytosolic Ca^2+^ signal during 0.5 Hz electrical stimulation-elicited Ca^2+^ transient. Black bars (40 pixels length) on the line scanning figure represent peak value of Ca^2+^ curves converted by these 40 pixel-width line scanning figure. *n* = 14 cells, ^∗∗^ *p* < 0.01. (b) FPL (1 *μ*M), a LTCC opener, induced an increase in cytosolic Ca^2+^ level. Nuclear Ca^2+^ increase had delayed kinetics in CICR disrupted (ryanodine pretreatment (Ry, 5 *μ*M)) hESC-VCMs, indicating that the nuclear Ca^2+^ delay was not caused by a possibly less CICR system in cell nuclei. Curves were normalized by scaling between 0 and 1. *n* = 12 cells, ^∗^ *p* < 0.05. (c) Diastolic Ca^2+^ in the cytosol or nucleus during 0.5 Hz or 2 Hz electrical stimulation. *n* = 9 cells, ^∗^ *p* < 0.05 or ^∗∗^ *p* < 0.01. (d) Ca^2+^ transient amplitude (ΔF/F0) in the cytosol or nucleus during 0.5 Hz or 2 Hz electrical stimulation. *n* = 9 cells, ^∗∗^ *p* < 0.01.

Figure 1(a) shows that the Ca^2+^ peaks (black bars, 40 pixels of width) were clearly separated into the cytoplasmic and nuclear groups, with a clear delay for the latter during electrical stimulation-induced Ca^2+^ transients, resembling those of _ext_Ca^2+^-induced. Although the onsets of cytosolic and nuclear Ca^2+^ signals upon electrical stimulation were virtually indistinguishable, both the rise and decay times of nuclear Ca^2+^ were significantly prolonged. Of note, no autonomous nuclear Ca^2+^ transients or waves could be observed in hESC-VCMs during pacing, distinctive from neonatal rat cardiomyocytes [24]. Furthermore, the application of the mitochondrial Ca^2+^ inhibitor CCCP and oligomycin did not prevent the delayed kinetics of nuclear Ca^2+^ signals (Figure S2). When hESC-VCMs were pretreated with ryanodine to block RyRs and thereby inhibiting all cytosolic and nuclear CICR, LTCC activation by FPL caused a dramatic cytosolic and nuclear Ca^2+^ rise (Figure 1(b)). Of note, FPL-induced nuclear Ca^2+^ rise exhibited delayed kinetics compared to FPL-elicited cytosolic Ca^2+^ rise. Taken together, these observations raise the possibility that nuclear Ca^2+^ rise is initiated by the diffusion of cytosolic Ca^2+^ and that the delayed kinetics could be attributed to differentially expressed sequestering proteins in the nucleus as diffusion barriers.

The slower kinetics of nuclear Ca^2+^ transient during low stimulation frequency (0.5 Hz) (Figure 1(a)) implied that nuclear Ca^2+^ transient would likely be more affected compared to cytosolic Ca^2+^ transient by higher stimulation frequency when the time available for Ca^2+^ removal is more restricted, resulting in an elevated diastolic Ca^2+^ level [25]. Therefore, we next examined the behavior of cytosolic and nuclear Ca^2+^ during high stimulation frequency at 2 Hz.  Figure 1(c) (and Figure S3) showed that increasing the stimulation frequency from 0.5 Hz to 2 Hz significantly increased the diastolic Ca^2+^ level without altering the systolic Ca^2+^ level in both the cytosol and nucleus. As a result, the Ca^2+^ transient amplitudes were dramatically decreased (Figure 1(d)). Interestingly, the effect of high stimulation frequency on the increase of diastolic Ca^2+^ and decrease of transient amplitude was significantly stronger on nuclear Ca^2+^ transient compared to that of cytosol (Figures 1(c) and 1(d)). This observation suggested that nuclear Ca^2+^ could be differentially regulated by the stimulation frequency.

### 3.2. Mechanistic and Structural Bases of Nuclear Ca^2+^ Signaling in hESC-VCMs

**Figure 2:**
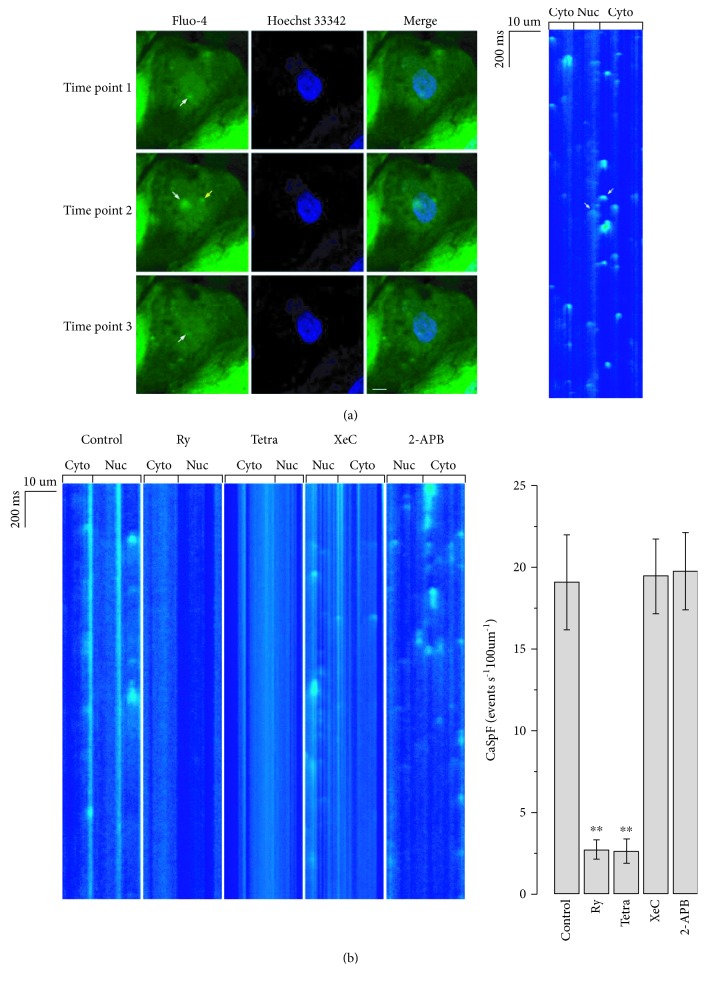
Nuclear Ca^2+^ sparks in hESC-VCMs. (a) Local nuclear Ca^2+^ signal could be observed in hESC-VCMs in both 2D (*left*) and line scanning imaging (*right*). White arrow showed Ca^2+^ sparks within the nucleus while yellow arrow indicated Ca^2+^ sparks that were near NE but did not fall into the nucleus area. Scale bar = 10 *μ*m. Time points for three sequential captures in 2D imaging are 0 sec, 9 sec, and 18 sec, respectively. (b) Local nuclear Ca^2+^ sparks were sensitive to ryanodine (Ry, 5 *μ*M) and tetracaine (Tetra, 1 mM), but not xestospongin C (XeC, 10 *μ*M) and 2-APB (10 *μ*M), pretreatment. *n* = 13 − 16 cells, ^∗∗^ *p* < 0.01.

For mechanistic insights, we investigated local Ca^2+^ sparks in hESC-VCMs as the elementary units.  Figure 2(a) shows representative time-lapsed 2D images and line-scanned results of nuclear Ca^2+^ sparks in a Hoechst-labeled nucleus. Pharmacologically, both cytosolic and nuclear Ca^2+^ sparks could be abolished by pretreatment with the RyR blocker ryanodine or tetracaine but were unaffected by the IP_3_R blocker Xec or 2-APB (Figure 2(b)).

**Figure 3:**
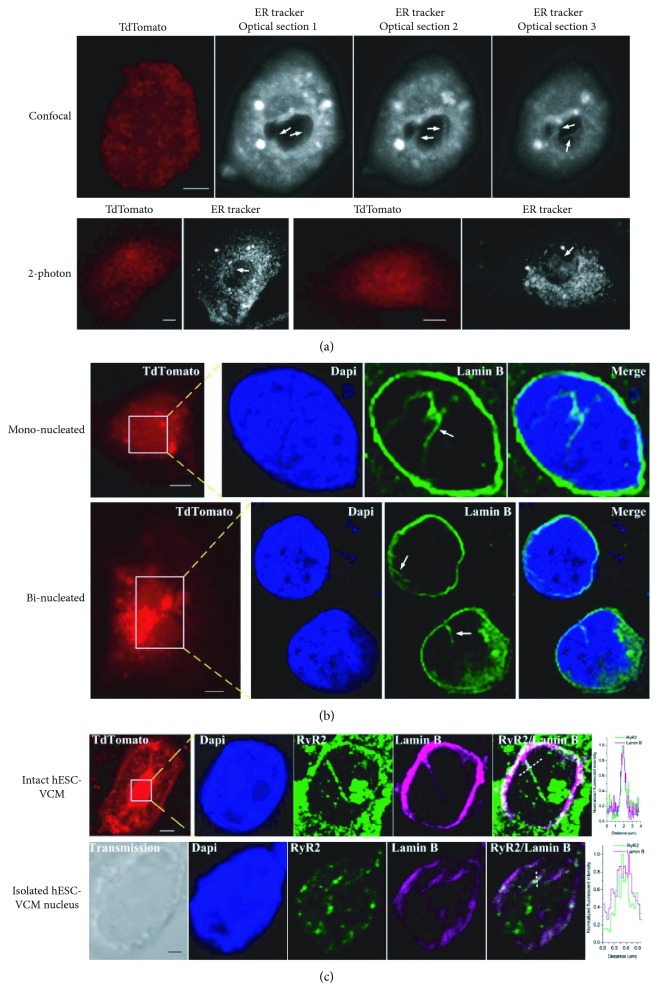
Nucleoplasmic reticulum (NR) structure in hESC-VCMs. (a) NR could be observed in ER tracker-loaded tdTomato-positive cells. The tdTomato fluorescence was driven by the ventricle-specific MLC2v promoter. Images were captured by a confocal (*upper*) or 2-photon (*lower*) microscope. Scale bar = 10 *μ*m. (b) Immunostaining by Lamin B (marker of NE) antibody revealed NR structure in both mononucleated (*upper*) and binucleated (*lower*) hESC-VCMs. The nuclear region indicated by the white box and yellow dashed lines was magnified. Scale bar = 10 *μ*m. (c) RyR2 localized to NR as revealed by coimmunostaining of RyR2 and Lamin B in intact cell (*upper*) and nucleus isolated from hESC-VCMs (*lower*). Graph shows fluorescence intensity for the indicated dashed line, highlighting the colocalization of RyR2 and Lamin B. Scale bar = 10 *μ*m for intact cells. Scale bar = 1 *μ*m for isolated nucleus.

**Figure 4:**
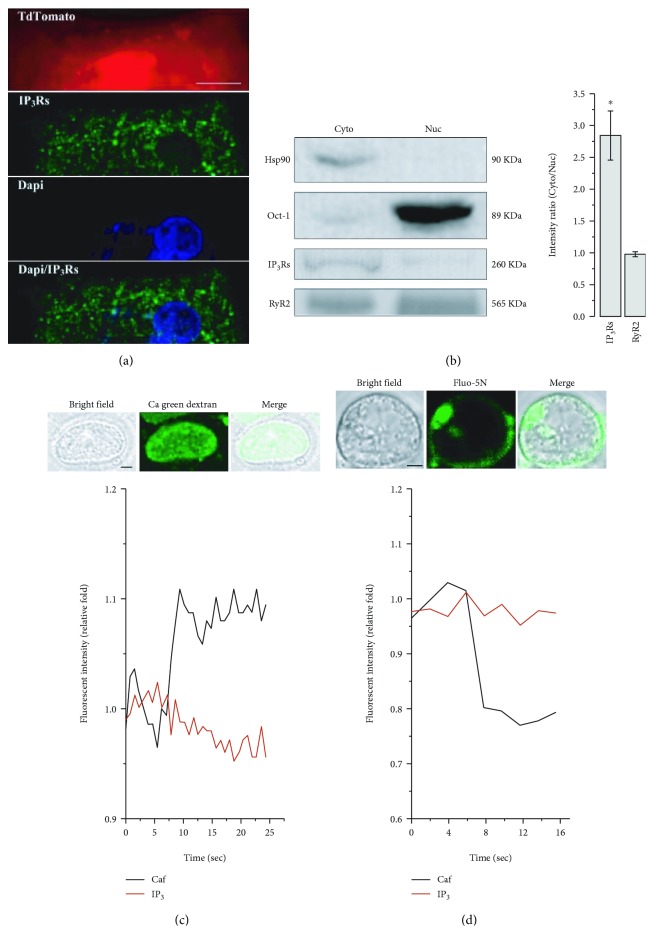
RyRs were responsible for nuclear Ca^2+^ release. (a) IP_3_Rs were mainly expressed in the cytosol in hESC-VCMs as revealed by immunostaining. Scale bar = 10 *μ*m. (b) Western blotting with equal amount of cytosolic and nuclear protein extracts showed IP_3_R proteins were mainly found in cytosol while RyRs were equally expressed in both cytosol and nuclei. Bar graph represents ratio of cytosolic band intensity to nuclear band intensity. (c) Isolated hESC-VCM nuclei were loaded with calcium green dextran with 10000 MW (*upper*), and caffeine (10 mM) but not IP3 (10 *μ*M) could elicit an increase in calcium green dextran fluorescence, indicating Ca^2+^ release into the nucleoplasm (*lower*). *n* = 20 − 30 nuclei. Scale bar = 2 *μ*m. (d) Isolated hESC-VCM nuclei were loaded with Fluo-5N AM (*upper*). Caffeine (10 mM) but not IP3 (10 *μ*M) decreased the dye fluorescence, showing Ca^2+^ release from nuclear envelope (*lower*). *n* = 14 − 18 nuclei. Scale bar = 2 *μ*m.

To investigate their structural basis, we performed confocal and 2-photon microscopy of ER-tracker-loaded and tdTomato-positive hESC-VCMs and discovered the presence of invaginations, termed nucleoplasmic reticulum (NR), from the nuclear envelope (NE) into the center of the nucleus (Figure 3(a)). Consistently, immunostaining by Lamin B antibody, an NE marker, likewise revealed the existence of NR in mono- as well as binucleated hESC-VCMs (Figure 3(b)). Coimmunostaining by Lamin B and RyRs further showed the localized expression of RyRs on NR (Figure 3(c)). By contrast, IP_3_Rs were primarily found in the cytosol (Figure 4(a)). Western blot analysis of the nuclear and cytosolic protein extracts, whose successful isolations were confirmed by the presence of the cytosolic marker Hsp90 and the nuclear protein Oct-1, respectively, validated the expression of RyR2 but not IP_3_Rs in the nucleus (Figure 4(b)). This is in contrast to results obtained with the same method in rat neonatal cardiomyocytes, where both RyR2 and IP_3_Rs are present in the nucleus [5]. To seek further functional evidence, we performed Ca^2+^ measurements on isolated intact hESC-VCM nuclei (Figure 4(c)) loaded with calcium green dextran which remained entrapped in the nucleoplasm after loading due to its molecular weight of ~10000 [17]. As anticipated from above, caffeine (by 110.5%, *p* < 0.01) but not IP_3_ (97.1%, *p* > 0.05) induced a significant increase in nucleoplasmic Ca^2+^. Consistently, Ca^2+^ measurement in nuclear envelop by Fluo-5N indicated that caffeine (by 82.6%, *p* < 0.01), but not IP_3_ (100.3%, *p* > 0.05), led to this Ca^2+^ release (Figure 4(d)). Collectively, our data showed that nuclear Ca^2+^ sparks are the elementary units of Ca^2+^ signal in hESC-VCMs which were mediated by RyRs expressed on the NE/NR.

### 3.3. Functional Consequences of Cytosol- and Nucleus-Specific Ca^2+^ Buffering

**Figure 5:**
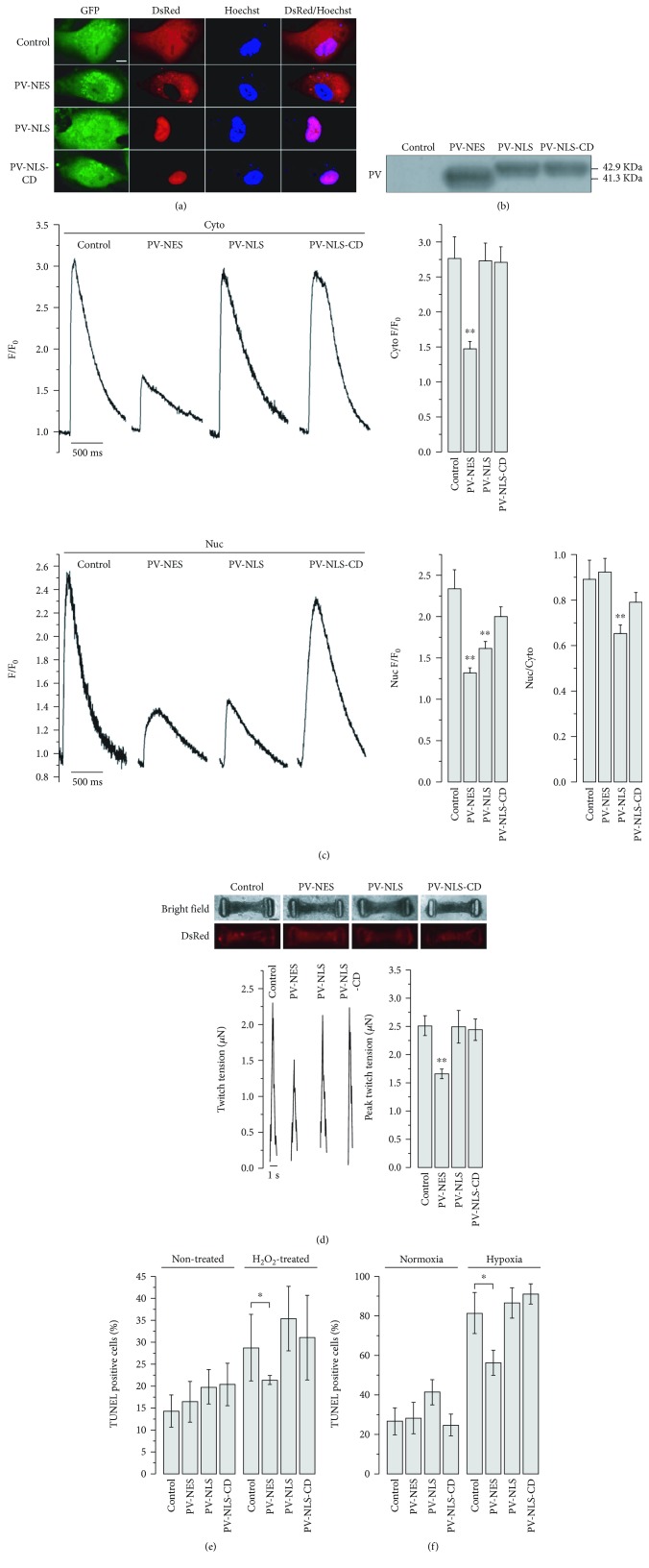
Expression of parvalbumin (PV) and its mutated form tagged with nuclear localization sequence (NLS) or nuclear export signal (NES). (a) Confocal imaging showed correct localization of PV fusion proteins. Scale bar = 10 *μ*m. (b) Western blot showing PV fusion proteins could be detected. (c) Cytosol-located PV expression (PV-NES for cytosolic Ca^2+^ buffering) dramatically diminished both cytosolic (*upper*) and nuclear (*lower*) Ca^2+^ transient during electrical pacing while nuclear PV expression only decreased the transient amplitude in nuclei without affecting cytosolic transient amplitude. PV-NLS-CD, the mutated form of PV, expression slightly, but not significantly, decreased nuclear Ca^2+^ transient amplitude. *n* = 10 − 27 cells, *p* < 0.01 (^∗∗^). (d) The effects of nuclear and cytosolic Ca^2+^ buffering on contractile force of hv-CMTs. *n* = 10 − 14 tissues, ^∗∗^ *p* < 0.01. Scale bar = 100 *μ*m. (e) TUNEL staining in hESC-VCMs showing hESC-VCMs treated with 100 *μ*M H_2_O_2_ for 1 hour. PV-NES expression caused a lower percentage of TUNEL-positive cells during H_2_O_2_ treatment compared to control (*p* = 0.05). Analysis was performed by 7 captures containing 150-200 cells. (f) During hypoxic condition (1% O_2_, 8 hours), PV-NES expression reduced the percentage of TUNEL-positive cells compared to control (*p* = 0.05). Analysis was performed by 3-5 captures containing 150-200 cells.

Parvalbumin (PV), a 9-11 kDa Ca^2+^ binding protein, has been used as an endogenous Ca^2+^ buffer [5, 6, 26, 27]. By tagging PV-DsRed with the nuclear localization sequence (NLS) or nuclear export signal (NES) and lentivirally delivering the fusion product [5], we attempted to selectively buffer free Ca^2+^ in the desired cellular compartment. We confirmed the expected subcellular localization patterns in GFP^+^ LV-MLC2v-GFP-transduced hESC-VCMs by confocal imaging and western blot analyses (Figures 5(a) and 5(b); 41.3 kDa for PV-NES, 42.9 kDa for PV-NLS, and 42.9 kDa for PV-NLS-CD). Functionally, cytosolic-specific PV expression (PV-NES) decreased both the cytosolic and nuclear Ca^2+^ peak amplitudes whereas nuclear-specific PV expression (PV-NLS) exclusively reduced that of nuclear but not cytosolic Ca^2+^ peaks. Transduction of a mutated form of PV with inactivated calcium binding sites targeted to the nucleus (PV-NLS-CD) had no effect. Interestingly, PV-NLS but not PV-NES altered the ratio of nuclear Ca^2+^ transient amplitude to that of the cytosol (Figure 5(c)), consistent with our observation that nuclear Ca^2+^ rise results from the diffusion of cytosolic Ca^2+^. Moreover, PV-NES diminished the contractile force of cardiac microtissues generated by hESC-CMs (hv-CMTs) (Figure 5(d)). In rat neonatal VCMs, nuclear Ca^2+^ buffering by PV has been reported to enlarge the nucleus size, translocate NFAT, and redistribute atrial natriuretic peptide (ANP) [5]. However, none of these was seen in hESC-VCMs (Figure S4), underscoring the significant species-specific difference. Action potential parameters were also not altered by PV transduction (Figure S5). We next found that cytosolic, but not nuclear, Ca^2+^ buffering protected hESC-VCMs from stimuli (H_2_O_2_ and hypoxia)-induced apoptosis (Figures 5(e) and 5(f)).

### 3.4. Nuclear Ca^2+^ Did Not Affect Pluripotency but the Functional Properties of Derived VCMs

To explore the role of nuclear and cytosolic Ca^2+^ in pluripotency and differentiation, we generated stably LV-PV-NLS- and LV-PV-NES-transduced hESC lines with persistent PV expression in the nucleus and cytosol, respectively (Figure S6A-B). Neither proliferation nor pluripotency was affected (Figure S6D-E). During spontaneous differentiation, a loss of pluripotency markers (Oct4, Nanog, and Sox2) as well as increased expression of three germ layers and cardiomyocyte markers could be seen (Figure S6G). Pluripotency and germ layer markers expressed at day 14 were chosen according to the expression pattern versus time in control hESCs. The markers for all three germ layers in 14-day-old embryoid body were present in all the PV groups (Figure S6C), indicating that calcium buffering did not alter pluripotency [28]. DsRed fluorescence could be continuously observed (Figure S6F) during the differentiation process. The differentiated hESC-VCMs displayed nuclear and cytosolic Ca^2+^ transient phenotypes with a pattern not different from those of postdifferentiation transduction (Figure S6H). These data suggest that nuclear and cytosolic Ca^2+^ did not grossly affect pluripotency and differentiation but the functional properties of their differentiated cardiac progeny.

### 3.5. Ca^2+^-Sensitive Pathways for Regulation of Gene Expression in hESC-VCMs

**Figure 6:**
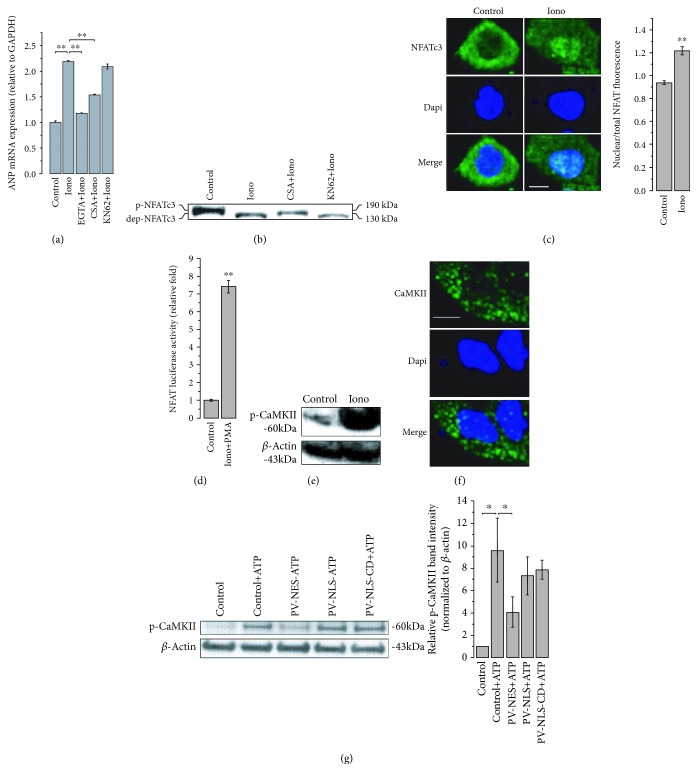
Pathways involved in Ca^2+^-regulated gene expression in hESC-VCMs. (a) As examined by Q-RT-PCR, treatment of ionomycin (2 *μ*M, 3 hours) dramatically increased mRNA level of ANP, which was blocked by EGTA (4 mM) and CsA (10 *μ*M), but not KN62 (10 *μ*M), pretreatment. ^∗∗^ *p* < 0.01. (b) As revealed by western blotting, ionomycin-induced Ca^2+^ elevation caused NFAT dephosphorylation which was blocked by the pretreatment of CsA (10 *μ*M), the calcineurin inhibitor, but not KN62 (10 *μ*M), the CaMK inhibitor. (c) Ionomycin treatment induced NFAT translocation from cytosol to nucleus. Bar graph showed the ratio of nuclear to total NFAT fluorescence. *n* = 33 − 36 cells, ^∗∗^ *p* < 0.01. Scale bar = 10 *μ*m. (d) Treatment of ionomycin (2 *μ*M) and PMA (100 ng/mL) significantly increased NFAT activity measured by luciferase assay. (e) Ionomycin treatment (2 *μ*M, 1 minute) caused CaMKII phosphorylation. (f) Immunostaining showed the cytosolic localization of endogenous CaMKII in hESC-VCMs. (g) ATP (100 *μ*M)-induced Ca^2+^ rise phosphorylated the endogenous CaMKII, which was blocked by the expression of cytosolic, but not nuclear, PV overexpression. *n* = 3, ^∗^ *p* < 0.05.

Next, we investigated the physiological role of nuclear Ca^2+^ in hESC-VCMs and the molecular pathways involved in its regulation. In rat neonatal cardiomyocyte, nuclear Ca^2+^ buffering causes nuclear enlargement via the activation of hypertrophic genes including ANP via calcineurin/NFAT activation [5]. We investigated whether hypertrophic response in hESC-VCMs, which has been shown to resemble fetal cardiomyocytes in maturity [29], could also be regulated by cytosolic/nuclear Ca^2+^. We investigated the role of both the calcineurin/NFAT pathway and the CAMKKII pathway as both have been indicated to modulate hypertrophic response in cardiomyocytes [30, 31]. As shown in Figure 6(a), the calcium ionophore ionomycin, which mediated Ca^2+^ entrance from extracellular environment, induced an increased expression of ANP, the cardiac hypertrophy marker. The effect of ionomycin was significantly blocked by EGTA (calcium chelator) pretreatment, indicating that it was Ca^2+^, rather than ionomycin itself, that caused the elevated expression of ANP. Moreover, this Ca^2+^-induced ANP expression was dramatically inhibited by CsA, the calcineurin (CN) inhibitor, but not altered by KN62, the CaMKII inhibitor. To further illustrate the functional CN/NFAT pathway in hESC-VCMs, western blotting using NFATc3 antibody was performed. The results indicated that ionomycin addition led to the shift of NFATc3 band to a lower molecular weight, suggesting the dephosphorylation of NFATc3, which was also blocked by CsA but not KN62 (Figure 6(b)). Immunostaining showed that ionomycin addition induced the translocation of NFATc3 from the cytosol to the nucleus (Figure 6(c)). Luciferase assay was performed to further demonstrate that NFAT in developmentally immature hESC-VCMs is functional (Figure 6(d)). CaMKII was also examined in hESC-VCMs and was found to be phosphorylated by ionomycin-induced Ca^2+^ elevation (Figure 6(e)). However, immunostaining showed that the endogenous CaMKII was mainly localized in the cytosol (Figure 6(f)) which is consistent with the limited role of CaMKII inhibition on Ca^2+^-induced gene expression. To further confirm the localization of CaMKII, PV Ca^2+^ buffering protein was used to block CaMKII phosphorylation caused by ATP, the Ca^2+^ mobilizer targeting purinergic receptors [32], and the result indicated that ATP-induced phosphorylation of endogenous CaMKII could be significantly diminished by PV expressed in cytosol but not nucleus (Figure 6(g)), which is consistent with the cytosolic localization of CaMKII.

### 3.6. Nuclear-Localized Ca^2+^-Sensitive CaMKII*δ*_B_ Overexpression in hESC-VCMs

**Figure 7:**
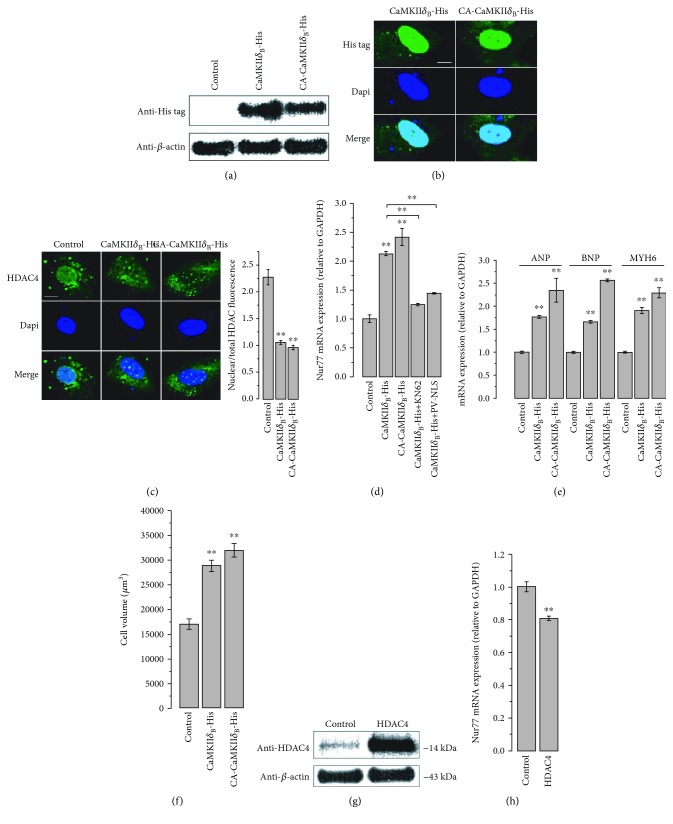
CaMKII*δ*B overexpression induced cardiac hypertrophy in hESC-VCMs. (a) Wild-type and constitutively active CaMKII*δ*B overexpression detected by western blotting. (b) The overexpressed CaMKII*δ*B targeted to cell nucleus. (c) Overexpression of CaMKII*δ*B led to nucleus to cytosol translocation of HDAC4. *n* = 20 − 30 cells, ^∗∗^ *p* < 0.01. (e) Overexpression of CaMKII*δ*B induced increased expression of Nur77, which was blocked by KN62 treatment (10 *μ*M) or PV-mediated nuclear Ca^2+^ buffering. ^∗∗^ *p* < 0.01. (e) Increased expression of cardiac hypertrophy markers (ANP, BNP, and MYH6) caused by CaMKII*δ*B overexpression. ^∗∗^ *p* < 0.01. (f) Cell volume, measured by CellTracker Green loading and confocal z-stacking, was increased by CaMKII*δ*B overexpression. *n* = 47 − 59 cells, *p* < 0.01 (^∗∗^). (g) Western blot showed HDAC4 overexpression. (h) HDAC4 overexpression led to decreased mRNA level of Nur77. ^∗∗^ *p* < 0.01.

Due to the lack of expression of endogenous CaMKII in hESC-VCM nucleus and the known role of nuclear-localized CaMKII*δ*_B_ on cardiac hypertrophy [33], we examined the effect of CaMKII*δ*_B_ overexpression in hESC-VCMs. 6X-histidine-tagged wild-type and constitutively active (CA) CaMKII*δ*_B_ were successfully expressed in hESC-VCMs as examined by western blotting (Figure 7(a)). Immunostaining showed that CaMKII*δ*_B_ targeted the cell nucleus (Figure 7(b)). Both wild-type and CA-CaMKII*δ*_B_ overexpression led to a nucleus to cytosol translocation of HDAC4 (Figure 7(c)) which is an endogenous inhibitor of the transcription factor MEF2. To further study the effect of CaMKII*δ*_B_ overexpression on MEF2-dependent gene transcription, the expression of Nur77, a known target of MEF2 [34, 35], was examined. CaMKII*δ*_B_ overexpression led to increased expression of Nur77 which could be blocked by either CaMKII inhibitor KN62 or nuclear Ca^2+^ buffering by PV (Figure 7(d)). This data indicated that CaMKII*δ*_B_ expression enhanced MEF2 activity by promoting HDAC4 translocation. We next examined whether enhanced MEF2 activity would lead to cardiac hypertrophy in hESC-VCMs. Indeed, qPCR results indicated increased expression of cardiac markers including ANP, BNP, and MYH6 (Figure 7(e)) and led to enlarged cell size (Figure 7(f)). However, the Ca^2+^ transient properties were not significantly changed by CaMKII*δ*_B_ overexpression (Figure S7). To further verify the regulatory role of HDAC4 on MEF2 activity, we found that HDAC4 overexpression in hESC-VCMs (Figure 7(g)) led to a decreased expression of Nur77 (Figure 7(h)). Collectively, nuclear Ca^2+^/CaMKII*δ*_B_/HDAC4/MEF2 pathway regulated cardiac hypertrophy in hESC-VCMs (Figure S9).

## 4. Discussion

The basis of how Ca^2+^ as a single second messenger can coordinate such diverse effects even within a single cell has been a longstanding topic of active investigation. Emerging evidence indicates that the spatial patterns of Ca^2+^ signals contribute to their specificity [6, 7, 36, 37]. Distinct from the well-established cytosolic Ca^2+^ signaling, nuclear Ca^2+^ signaling has been shown to likewise affect a range of cellular processes from gene transcription [8, 9] to cell proliferation [6, 38] as well as CM function [5, 39]. Cytosolic Ca^2+^ is largely stored in the endoplasmic reticulum (ER, or SR of muscle cells); the nuclear envelope (NE) is composed of an outer membrane contiguous with the ER and an inner membrane separated by a luminal space that serves as a Ca^2+^ store (Figure S9). Depending on the specific cell type, nuclear Ca^2+^ is regulated by (1) Ca^2+^ entry into the nucleoplasm by passive diffusion from cytosol through nuclear membrane pores [40]; (2) direct Ca^2+^ release from the inner membrane of NE into the nucleoplasm [41]; and (3) the nucleus has its own reticular network that is continuous with ER and NE [42, 43], the so-called nucleoplasmic reticulum (NR, a.k.a. nuclear tubules), invaginations that reach deeply to increase the surface-to-volume ratio for facilitated Ca^2+^ entry and exit. NR has been visualized by two-photon microscopy in a variety of mammalian cells including CMs [5, 44], with RyR and IP_3_R as well as phospholamban (PLB) expressed on the NR membrane [45–47], allowing nuclear Ca^2+^ to regulate cellular functions independently of cytosolic Ca^2+^ change [6, 48]. Our presented data show for the first time that NR is also present in hESC-VCMs and that nuclear Ca^2+^ signaling is functional in hESC and hESC-VCMs.

We found that Ca^2+^ could diffuse to hESC-VCM nucleoplasm from cytosol, resulting in a delayed kinetics of nuclear signals which is not dye-specific and not due to different Ca^2+^ dye characteristics in the nucleus and the cytosol (Figure S2). This is also consistent with a recent publication indicating that cytosolic Ca^2+^ rise during EC coupling diffused into the nucleus, resulting in the slower rise time of nuclear Ca^2+^ transient and a slower decay time due to the fact that the SERCA2 located on NE, was mainly facing the cytosol, and nuclear Ca^2+^ had to diffuse out of the nucleus to be pumped back to the NE [25]. More importantly, due to this slower kinetics of nuclear Ca^2+^ transients, high stimulation frequency (2 Hz) was able to differentially regulate nuclear Ca^2+^: compared to cytosolic Ca^2+^ transient, nuclear Ca^2+^ transients turned into a more sustained (higher diastolic Ca^2+^) Ca^2+^ signal with lower fluctuation (lower transient amplitude) by high stimulation frequency in hESC-VCMs. The functions of this frequency-dependent modulation of nuclear Ca^2+^ signal remain to be determined [25].

In murine and human ESC-CMs, it has been suggested that spontaneous Ca^2+^ transients are triggered by IP_3_-mediated Ca^2+^ release, which is then amplified and modulated by RyR-mediated Ca^2+^ release [49–51]. Indeed, IP_3_R is highly expressed in conductive CMs in either embryonic or adult hearts [50]. Developmentally, at the early embryonic or fetal stage, the heart has not yet formed or its contractile function for pumping blood is not yet necessary. Therefore, the main function of IP_3_ has been theorized to increase intracellular Ca^2+^ for directing developmental pathways rather than for EC coupling. This is supported by the preferential intra and perinuclear location of IP_3_R for nuclear signaling and IP_3_R downregulation after birth [52, 53]. Our experiments revealed that nuclear Ca^2+^ of hESC-VCMs is regulated only by RyRs, like in the SR, but not IP_3_R. It is reported that IP_3_Rs in ventricular cardiomyocytes are around 5-6 times less abundant than those in atrial cells [54–56], which may also help explain the limited role of IP_3_R in nuclear Ca^2+^ signal since only ventricular cardiomyocytes derived from stem cells are used in this study.

In neonatal rat VCMs, buffering nuclear Ca^2+^ reduces the global Ca^2+^ transient amplitude (by ~50%) [5]. In adult VCMs, nuclear but not cytosolic Ca^2+^ buffering prolongs APD, suggesting that nuclear Ca^2+^ is specifically responsible for electrophysiological remodeling. To correlate cytosolic or nuclear Ca^2+^ changes early on during pluripotency or late after ventricular specification to phenotypic properties of hESC-VCM, we attempted to regulate the Ca^2+^ levels in different cell compartments by targeted expression of calcium buffer protein PV. PV targeted to the cytosol (PV-NES) diminished *both* cytosolic and nuclear Ca^2+^ rise elicited electrically because any reduced cytosolic Ca^2+^ increase would decrease the contribution of passive diffusion to nuclear Ca^2+^ increase (cf. isolation of active release and diffusion components in our experiments with intact cells and isolated nuclei provides a basis for interpretation here). At the physiological level, cytosolic Ca^2+^, but not nuclear Ca^2+^, blockade prevented stimuli-induced apoptosis (Figures 5(e) and 5(f)). PV-CD, with a weakened buffering capacity, led to intermediate effects between PV and controls. Indeed, a Ca^2+^(dose)-dependent relationship was observed (see Figure S8).

Cytosolic/nuclear Ca^2+^ also regulates gene transcription in hESC-VCMs. In contrast to rat neonatal cardiomyocytes, our data indicated that an increase of ANP expression induced by elevation of cytosolic/nuclear Ca^2+^ level was mediated by CN rather than CaMKII. CN is known as calcium-dependent serine-threonine phosphatase. By dephosphorylation, CN activates nuclear factor of activated T-cells (NFAT) and causes NFAT translocation from cytosol to nucleus to modulate gene transcription. Numerous publications indicate the connection between CN/NFAT pathway and cardiac hypertrophy. For instance, in mice, CN expression level and activity are increased during early pregnancy, which may be caused by the pregnancy-associated sex hormone progesterone. This enhanced expression and activity of CN increase NFAT activity and lead to cardiac hypertrophy during pregnancy. Moreover, CsA treatment blocks pregnancy-induced hypertrophy in mice [57], which is consistent with the notion that CN-dependent cardiac remodeling can be reversed [30]. In hESC-VCMs, we similarly found that NFAT dephosphorylation and translocation could be induced by Ca^2+^, indicating a functional CN/NFAT pathway in hESC-VCMs. On the other hand, CaMKII is a serine/threonine-specific protein kinase that can be regulated by Ca^2+^/calmodulin complex. CaMKII is well known to be involved in cardiac hypertrophy and heart failure [31]. Indeed, it is reported that cardiac-specific nuclear CaMKII*δ*_B_ induces hypertrophy [33]. During cardiac hypertrophy, the cell nucleus-localized CaMKII plays its roles in histone H3 phosphorylation and chromatin remodeling [58]. Because of the important role of CaMKII in hypertrophy, CaMKII is deemed a useful target in the clinical treatment of cardiac hypertrophy and remodeling [59]. Although the endogenous CaMKII could be phosphorylated by Ca^2+^ in hESC-VCMs, CaMKII was mainly expressed in cytosol, which explains its limited role in gene transcription in hESC-VCMs. However, when we overexpressed the nucleus-targeted isoform CaMKII*δ*_B_ in hESC-VCMs, we found that CaMKII*δ*_B_ could indeed induce cardiac hypertrophy through nuclear Ca^2+^/CaMKII*δ*_B_/HDAC4/MEF2 pathway (Figure 7 and Figure S9).

## 5. Conclusions

Overall, we sought to develop a cause-and-effect relationship between any changes in nuclear or cytosolic Ca^2+^ signals and the corresponding biological responses. We conclude that (1) nuclear Ca^2+^ signal has delayed kinetics compared to cytosolic Ca^2+^ signal during global Ca^2+^ changes like waves or transients, indicating that nuclear Ca^2+^ is initiated by Ca^2+^ diffusion from cytosol and (2) local nuclear Ca^2+^ sparks in hPSC-VCMs are mediated by RyRs, and NR serves as structural basis. In addition, PV-mediated Ca^2+^ buffering can successfully modulate Ca^2+^ transient and stimuli-induced apoptosis in hPSC-VCMs. Moreover, the endogenous cytosolic Ca^2+^/CN/NFAT pathway is functional in hESC-VCMs, and the overexpression of nucleus-localized CaMKII*δ*_B_ led to cardiac hypertrophy through nuclear Ca^2+^/CaMKII*δ*_B_/HDAC4/MEF2 pathway. Collectively, these findings provide the insights of nuclear Ca^2+^ signal in hPSC-VCMs, which may lead to novel strategies for maturation as well as improved systems for disease modeling, drug discovery, and cell-based therapies.

## Data Availability

The data used to support the findings of this study are included within the supplementary information files.

## Supplementary Materials

Supplementary Materials Figure S1: nuclear Ca^2+^ signaling during Ca^2+^ waves. Figure S2. Figure S3: illustrative diagram of the differential effect of high stimulation frequency on cytosolic and nuclear Ca^2+^ transient. Figure S4. Figure S5: the effects of nuclear and cytosolic Ca^2+^ buffering on hESC-VCM action potential. Figure S6: expression of parvalbumin (PV) tagged with nuclear localization sequence (NLS) or nuclear export signal (NES) in hESC. Figure S7: CaMKII*δ*B overexpression did not alter Ca^2+^ transients in hESC-VCMs. Figure S8: higher DsRed expression level was correlated to lower Ca^2+^ transient peak in PV-NES expressing hESC-CMs. Figure S9: schematic diagram of cytosolic/nuclear Ca^2+^ signaling in hESC-VCMs.
