# Supplementary material for: Structural and Mechanistic Bases of Nuclear Calcium Signaling in Human Pluripotent Stem Cell-Derived Ventricular Cardiomyocytes
Source: Stem Cells Int. 2019 Apr 1;2019:8765752. doi: 10.1155/2019/8765752 (PMC6466844; doi:10.1155/2019/8765752)

**Supplementary Materials**

**
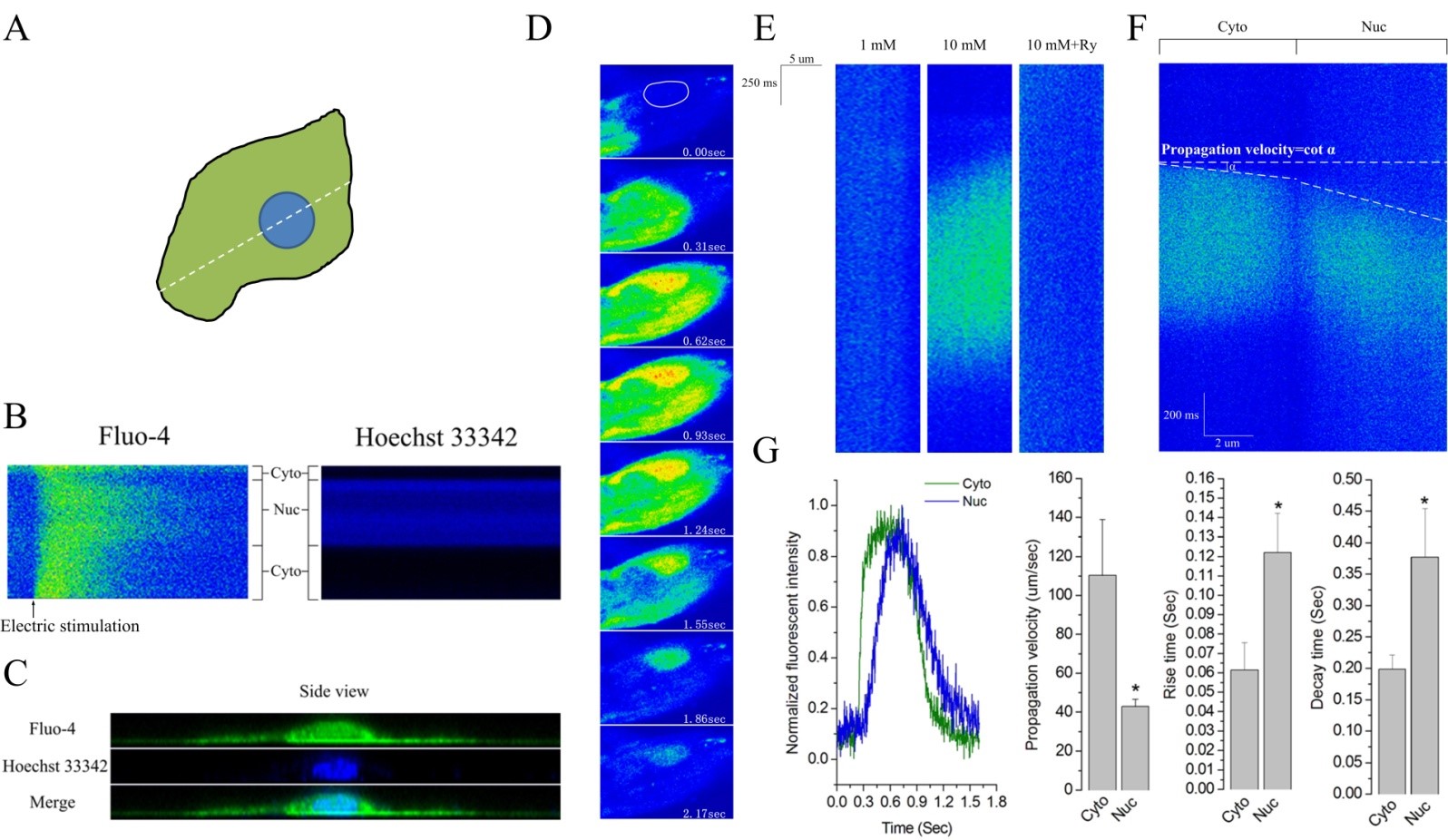
**

**Figure S1. Nuclear Ca^2+^ signaling during Ca^2+^ waves**. (A) Line selected for nuclear Ca^2+^ signal measurement. (B) After recording Ca^2+^ signal (*left*), Hoechst fluorescence was recorded (*right*) by line‐scanning in order to accurately mark nuclear region. (C) Z‐stacking image showed the center region of the cell was occupied by the nucleus. (D) A propagating Ca^2+^ wave in hESC‐VCMs incubated in high external Ca^2+^ (10 mM) buffer. The nucleus was marked by a white circle. (E) In line‐scanning recording, Ca^2+^ wave could be observed in high external Ca^2+^ buffer, and was sensitive to ryanodine pretreatment (Ry, 5 µM). (F) Nuclear Ca^2+^ signal had slower propagating velocity compared with cytosolic Ca^2+^ signal. (G) Converted from line‐scanning figure, Ca^2+^ curve from nucleus showed a delayed kinetics compared to cytosolic Ca^2+^ signal. Curves were normalized by scaling between 0 and 1. Propagating velocity was calculated as the ratio of propagating distance to propagating time. n=12 cells, * p<0.05.

**
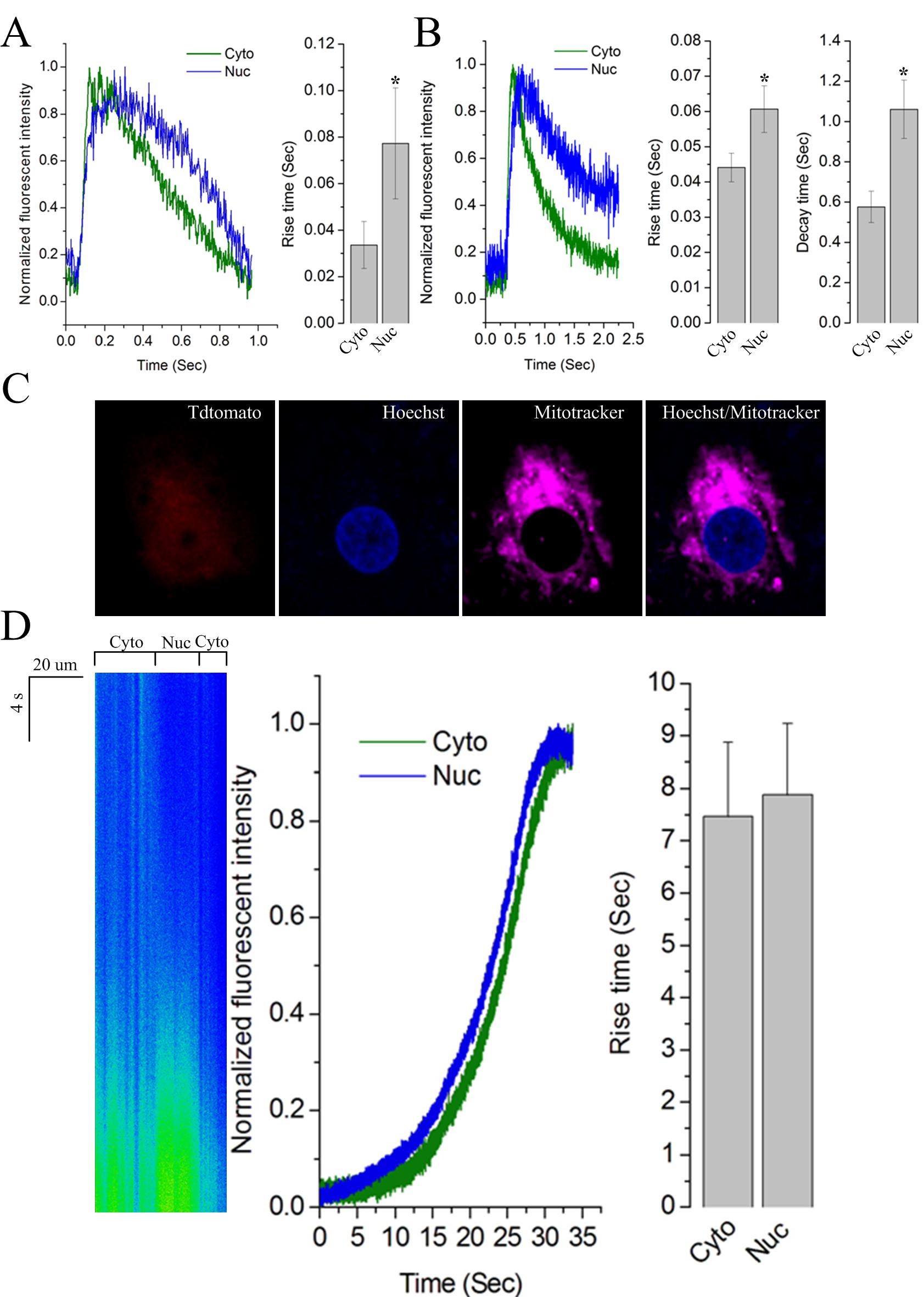
Figure S2.** (A) Electrical stimulation-induced Ca^2+^ transient with pretreatment of mitochondrial Ca^2+^ inhibitors CCCP (10 µM) and oligomycin (10 µM). n=7 cells, *p*<0.05 (*). (B) Electrical stimulation-induced Ca^2+^ transient measured by X-Rhod-1. n=7 cells, *p*<0.05 (*). (C) Cell nucleus was marked by mitotracker deep red. (D) Simultaneous uncaging of caged‐Ca^2+^ (NP‐EGTA) and Ca^2+^ recording was performed in ling‐scanning mode, and no significant difference of Fluo‐4 kinetics was obtained in nuclear or cytosolic region. n=8 cells.

**
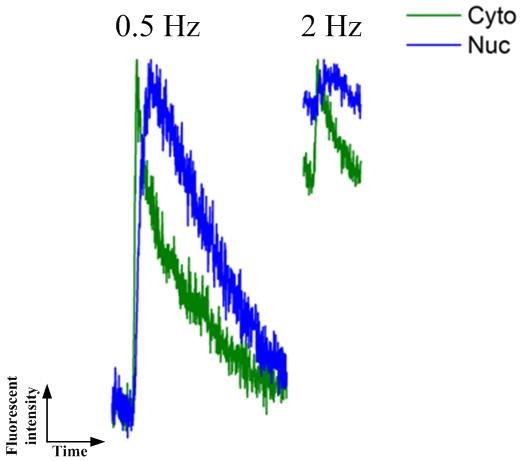
**

**Figure S3. Illustrative diagram of the differential effect of high stimulation frequency on cytosolic and nuclear Ca^2+^ transient.**


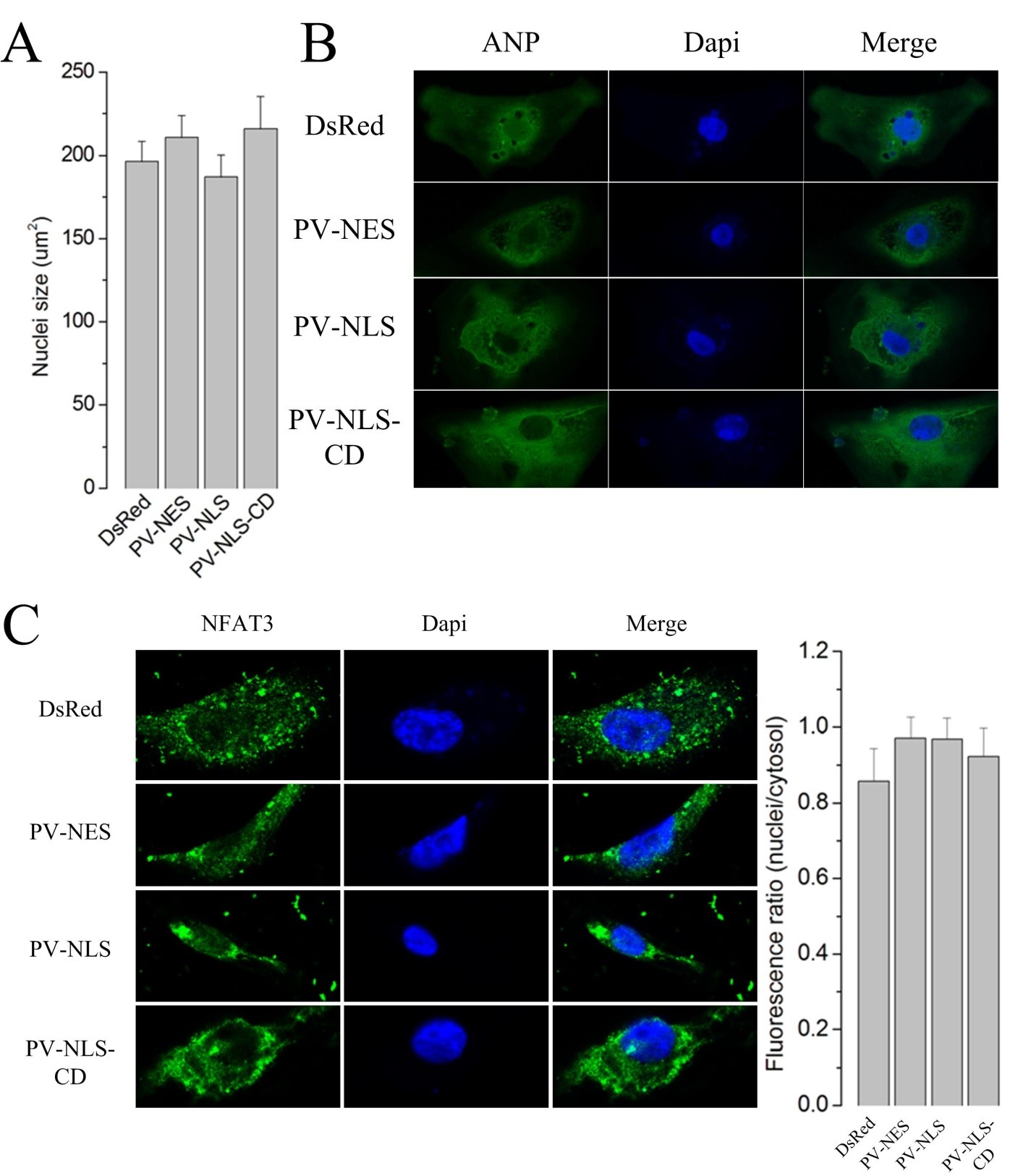


**Figure S4.** (A) Nuclear Ca^2+^ buffering failed to change nuclear size of hESC-VCMs. n=56-78 nuclei. (B) Nuclear Ca^2+^ buffering failed to change ANP pattern. (C) Nuclear Ca^2+^ buffering failed to change NFAT pattern. n=10 to 12 cells.


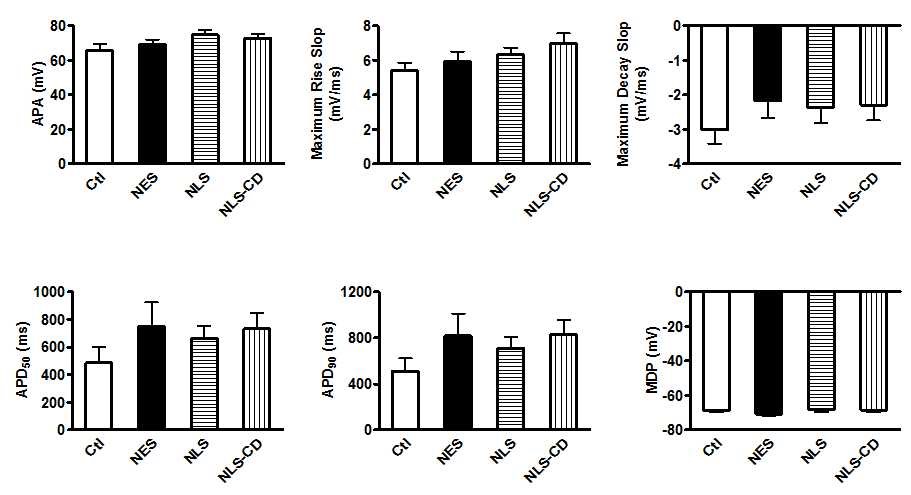


**Figure S5. The effects of nuclear and cytosolic Ca^2+^ buffering on hESC-VCMs action potential.** n=7 to 12 cells.

**
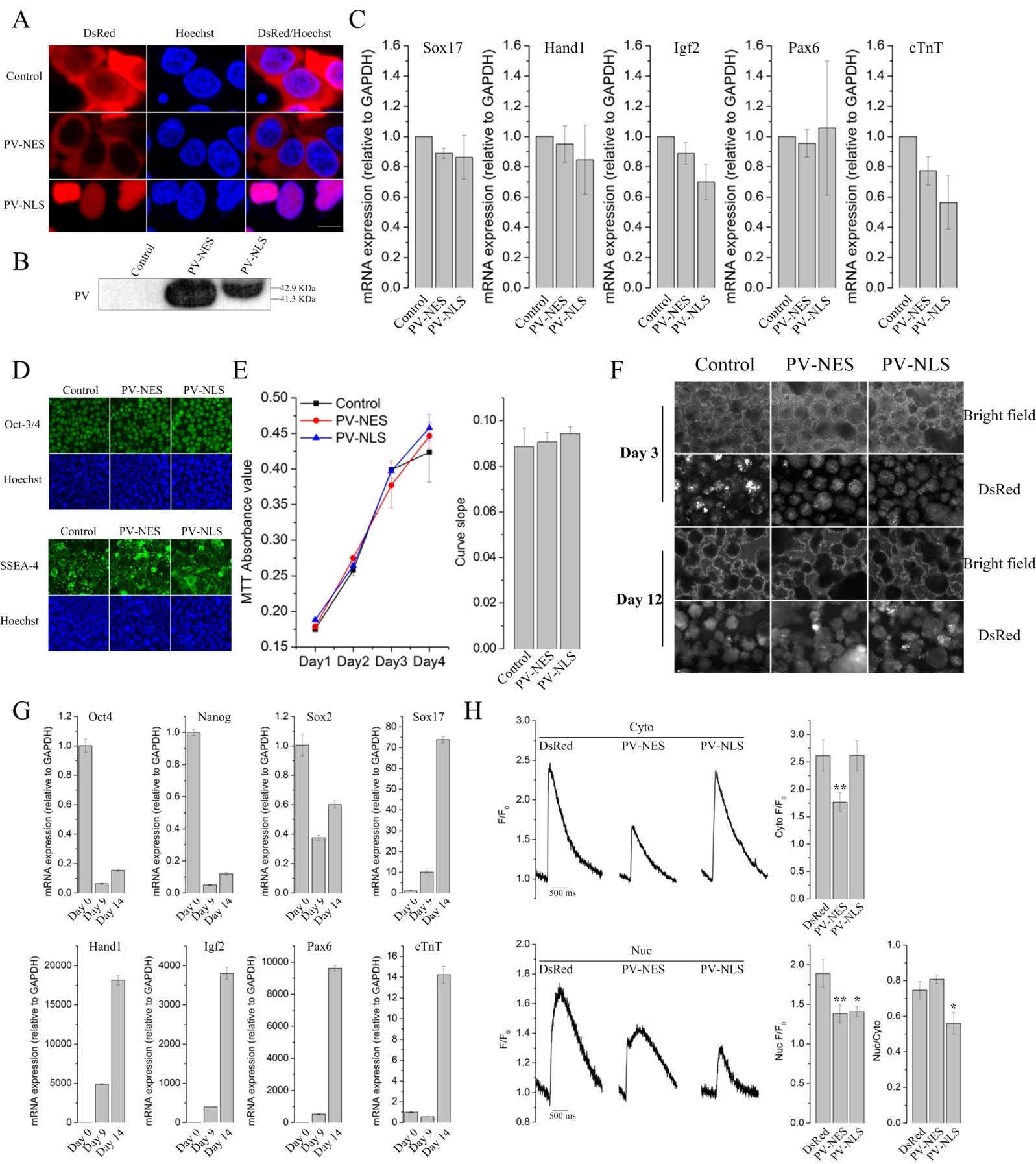
Figure S6. Expression of parvalbumin (PV) tagged with nuclear localization sequence (NLS) or nuclear export signal (NES) in hESC.** (A) Confocal imaging showed the correct localization of the PV fusion proteins. Scale bar=10 µm. (B) Western blot showing PV fusion proteins could be detected. (C) Effect of nuclear/cytosolic Ca^2+^ buffering on the expression of 3-germ layers makers (Sox17 for endoderm, Hand1 and Igf2 for mesoderm, Pax6 for ectoderm and cTnT for cardiomyocytes) in Day 14 embryoid body. (D) Pluripotency markers (Oct-3/4 and SSEA-4) of stem cells were not altered after expressing nuclear and cytosolic PV protein. (E) Proliferation rate of stem cells was not changed after PV expression. (F) DsRed fluorescence could be detected during spontaneous differentiation process of PV-expressing stem cells. (G) During the differentiation process, a loss of pluripotency markers (Oct4, Nanog and Sox2) and increased expression of 3-germ layers and cardiomyocyte makers could be observed. Figure shows expression pattern in control cells. (H) Cytosolic PV diminished both cytosolic and nuclear Ca^2+^ rise while nuclear PV only decreased nuclear Ca^2+^ rise without altering cytosolic transient during 0.5 Hz electrical stimulation in cardiomyocytes differentiated from stem cells stably expressing PV protein. n=5-16 cells, *p<0.05 or **p<0.01.

**
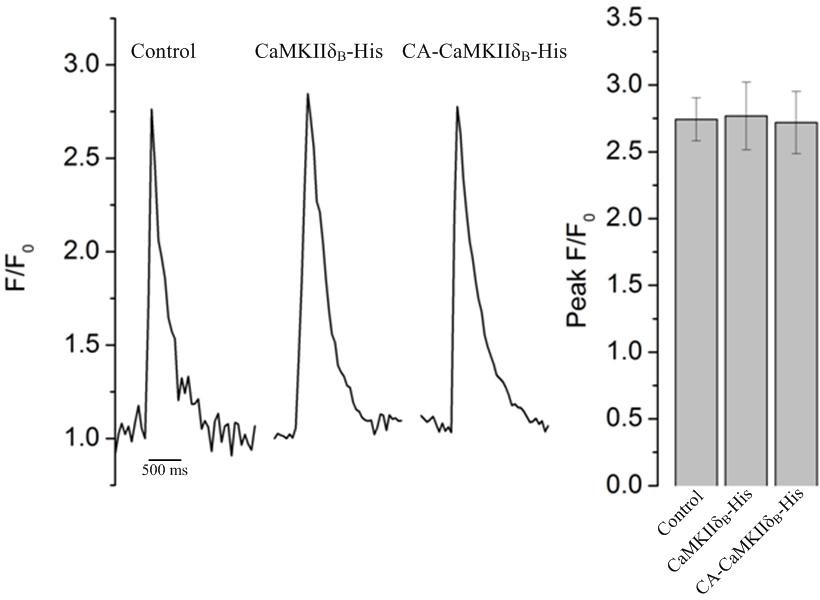
**

**Figure S7. CaMKIIδ_B_ overexpression didn't alter Ca^2+^ transients in hESC-VCMs. n=7 to 10 cells.**

**
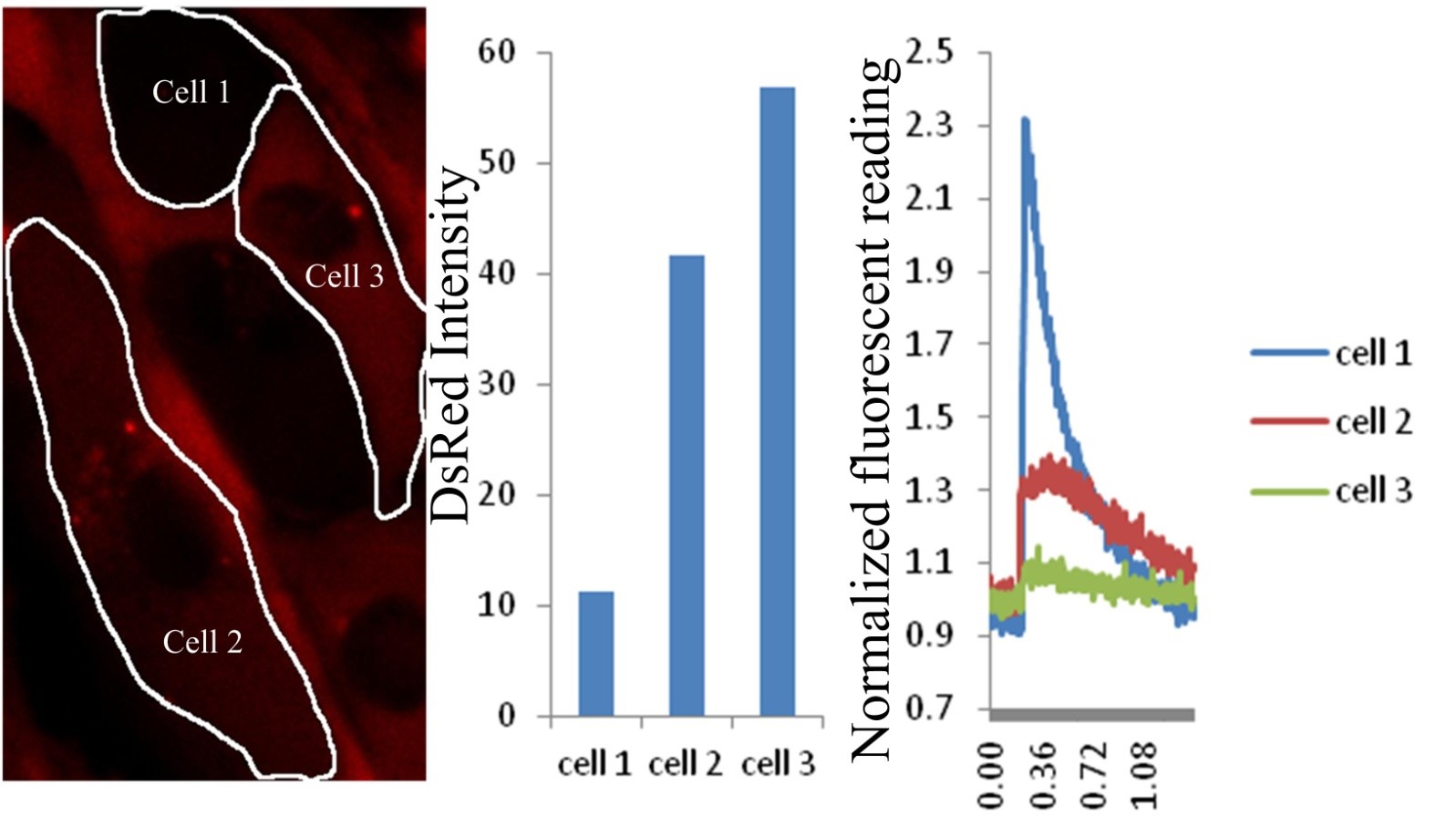
**

**Figure S8. Higher DsRed expression level was correlated to lower Ca^2+^ transient peak in PV-NES expressing hESC-CMs.**

**Figure S9. Schematic diagram of cytosolic/nuclear Ca^2+^ signaling in hESC-VCMs.**


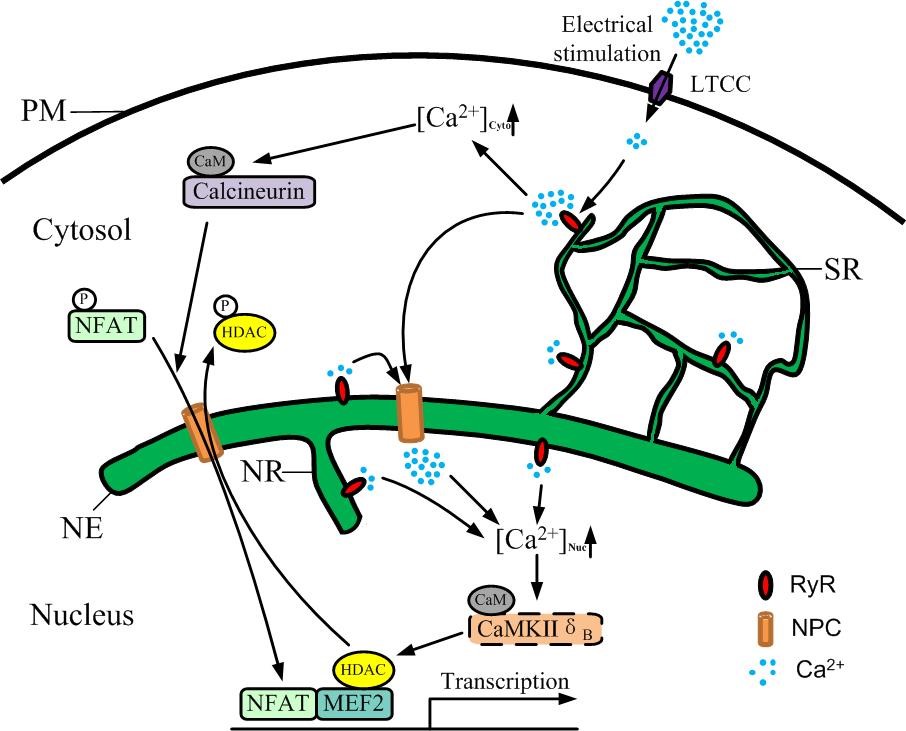

Supplement: Supplementary Materials — Figure S1: nuclear Ca2+ signaling during Ca2+ waves. Figure S2. Figure S3: illustrative diagram of the differential effect of high stimulation frequency on cytosolic and nuclear Ca2+ transient. Figure S4. Figure S5: the effects of nuclear and cytosolic Ca2+ buffering on hESC-VCM action potential. Figure S6: expression of parvalbumin (PV) tagged with nuclear localization sequence (NLS) or nuclear export signal (NES) in hESC. Figure S7: CaMKIIδB overexpression did not alter Ca2+ transients in hESC-VCMs. Figure S8: higher DsRed expression level was correlated to lower Ca2+ transient peak in PV-NES expressing hESC-CMs. Figure S9: schematic diagram of cytosolic/nuclear Ca2+ signaling in hESC-VCMs. [file 8765752.f1.docx]
